# The mediating role of depression in the association between health-related quality of life and suicidal ideation in adolescents: findings from a longitudinal study

**DOI:** 10.3389/frcha.2025.1567387

**Published:** 2025-08-14

**Authors:** Martina Preisig, Isabelle Häberling, Lukasz Smigielski, Sophie Emery, Noemi Baumgartner, Mona Albermann, Michael Strumberger, Klaus Schmeck, Lars Wöckel, Suzanne Erb, Bruno Rhiner, Brigitte Contin-Waldvogel, Susanne Walitza, Gregor Berger

**Affiliations:** ^1^ Clinic for Child and Adolescent Psychiatry and Psychotherapy, Psychiatric University Hospital Zurich, Zurich, Switzerland; ^2^ Outpatient Psychology Wil, Psychiatric Hospital St. Gallen Nord, Wil, Switzerland; ^3^ Outpatient Clinic, Psychiatric Services Lucerne, Lucerne, Switzerland; ^4^ Department of Clinical Research, University of Basel, Basel, Switzerland; ^5^ Center for Child and Adolescent Psychiatry and Psychotherapy, Clienia Littenheid AG, Littenheid, Switzerland; ^6^ Special Offers and Projects, Child and Adolescent Services St. Gallen, St. Gallen, Switzerland; ^7^ Child and Adolescent Psychiatry, Psychiatric Services Thurgau, Weinfelden, Switzerland; ^8^ Child and Adolescent Psychiatry, Psychiatric Services Baselland, Liestal, Switzerland; ^9^ Neuroscience Center Zurich, University of Zurich and ETH Zurich, Zurich, Switzerland; ^10^ Zurich Center for Integrative Human Physiology, University of Zurich, Zurich, Switzerland

**Keywords:** health-related quality of life, HRQoL, suicidality, suicidal ideation, depression, children, adolescents

## Abstract

**Introduction:**

Adolescent suicidality is a significant public health issue. To develop effective interventions aimed at preventing suicide in this vulnerable population, it is essential to understand the complex interplay of health-related quality of life, depression and suicidal ideation.

**Methods:**

For this purpose, we analyzed longitudinal data of 250 children and adolescents diagnosed with major depressive disorder (M = 15.7, SD = 1.6, range 8–18 years, 74% females). The main goal of the study was to examine whether the effect of health-related quality of life on individual trajectories of suicidal ideation is mediated by depression severity. A series of t-tests, Chi-squared-tests, Fisher’s exact tests and a mediation analysis including three robust linear mixed-effects models were conducted.

**Results:**

Depressed adolescents with suicidal ideation reported lower health-related quality of life across physical, psychological, peer, and school domains compared to those without suicidal ideation, while no significant difference was observed in the family-related domain. Psychological well-being emerged as the sole domain of health-related quality of life with a direct influence on suicidal ideation. Notably, depression severity mediated the effect of physical, psychological, peer- and school-related quality of life on suicidal ideation.

**Discussion:**

Our findings suggest that improving health-related quality of life reduces depressive symptoms, which in turn leads to lower suicidal ideation. This highlights the importance of including health-related quality of life in the clinical assessment of suicide risk as well as targeting health-related quality of life in therapeutic interventions. In the light of the results of this study, interventions should not only focus on classical clinical criteria of psychiatric diagnoses such as major depressive disorder, but also on broader, more resource-oriented constructs such as health-related quality of life to better mitigate the risk of suicide in this vulnerable population.

**Clinical Trial Registration:**

www.ClinicalTrials.gov, identifier [NCT03167307].

## 1 Introduction

Suicidality poses a highly relevant public health concern, particularly in adolescents. In this population, suicide ranks as the fourth leading cause of death globally (1–3). Previous research has shown that health-related quality of life (HRQoL) is inversely associated with psychiatric symptoms including those typically observed in depression, such as suicidality (4–8). However, the intricate relationship between HRQoL, depression, and suicidal ideation in children and adolescents, particularly the mediating effects of depression on the impact of HRQoL on suicidal ideation, remains poorly understood.

HRQoL encompasses the physical, emotional, social, and psychological aspects of an individual's subjective well-being and functioning (9). It is often assessed across multiple dimensions such as physical well-being, psychological well-being, autonomy and parent relation, peers and social support, and school environment, reflecting the multidimensional nature of health (9). There are several advantages of HRQoL over traditional measures of mental health. HRQoL adds additional information to measures by also assessing differences in individuals with low symptomatology (10). As a continuous measure, HRQoL tends to provide greater differentiation between different levels of mental health than traditional categorical diagnostic criteria with strict cut-off values (11). Through its subjective, self-reported assessment and independency of specific diagnoses, HRQoL is comparable across different types of patients and is transdiagnostic in nature (11). While HRQoL and psychiatric symptoms are associated, they are not always linearly correlated (8, 10, 12). Treatment of depression generally improves HRQoL, however HRQoL tends to change more slowly than psychiatric symptoms and only up to half of the change in HRQoL can be explained by improvement in depression symptoms (8, 12). For the above-mentioned reasons, assessing and targeting HRQoL should be an important part of treating depression and HRQoL should be considered as an alternative primary outcome when testing for treatment efficacy in clinical trials (12).

In both general and adolescent populations, depressive symptoms and HRQoL are inversely related (10, 13–15). The relationship between suicidality and HRQoL has been increasingly studied in adult populations in the last decade (11, 16, 17). Researchers generally agree that lower HRQoL is associated with increased suicidal thoughts and behaviors as well as self-harm. A recent systematic review by Le et al. (16) shows that adolescents with non-suicidal self-harming behavior as well as those who attempted suicide have lower HRQoL than those without a history of these behaviors. However, no study to date has assessed the relationship between suicidal ideation and HRQoL in a sample of depressed adolescents (16). The mentioned systematic review also highlights the lack of longitudinal studies on HRQoL in depressed adolescents, especially those with major depressive disorder (MDD).

The mediating role of depression in the relationship between HRQoL and suicidal ideation remains even less explored. Understanding this effect is crucial for developing interventions aimed at improving HRQoL and reducing depressive symptoms, thereby mitigating the risk of suicide in the vulnerable population of children and adolescents. Numerous studies have highlighted the mediating role of depression in the relationship between various psychological factors and suicidal ideation (18–28). In adult populations and cross-sectional designs, depression has been found to mediate the relationship between emotional stability (28), impulsivity (18), psychological strain (27), gratitude (24) and suicidal ideation. In a population of 250 adult cancer patients in Nigeria, HRQoL was found to be indirectly associated with suicidal ideation through psychological distress, whereas no direct effect of HRQoL on suicidal ideation was found (19). In adolescent populations, depression has been identified as a mediator between parent-adolescent conflict (25) as well as school bullying (21) and suicidal ideation in cross-sectional studies. In pregnant female adolescents in Colombia, depression was found to mediate the effect of HRQOL measured by the Kidscreen-52 (9) on suicidal ideation (26). These studies suggest a mediating effect of depression between various psychological factors and suicidality, as well as between HRQoL itself and suicidality. To our knowledge, no study to date has assessed the relationship between HRQoL, depression and suicidal ideation with a focus on the mediating role of depression in a longitudinal, clinical sample of children and adolescents in the cultural context of European countries.

In the present study, we used a longitudinal dataset of 250 children and adolescents diagnosed with MDD from the longitudinal Omega-3 depression study (29) to test whether the effect of HRQoL on individual trajectories of suicidal ideation is mediated by depression severity. By examining data at three time points, we aimed to better understand the temporal dynamics and interactions between these three variables. Based on existing literature and the conceptual framework outlined above, our first hypothesis was that HRQoL is lower in depressed adolescents with suicidal ideation than in depressed adolescents without suicidal ideation. Our second hypothesis was that depression severity mediates the effect of HRQoL on suicidal ideation for each of the five domains of the KidScreen-27 (9). In line with the study by Soto-Chavarría et al. (26) and further studies outlined above, this hypothesis implies that lower HRQoL leads to higher levels of depression, which in turn increases the likelihood of suicidal ideation. By testing these hypotheses, we aimed to elucidate the pathways through which HRQoL influences suicidal thoughts, providing valuable insights for the development of effective mental health intervention and suicide prevention for children and adolescents.

## 2 Methods

The data used for this study stems from a phase III, 36-week multi-center double-blind placebo-controlled clinical trial investigating the effect of Omega-3 fatty acids on pediatric depression (29). The trial took place from 28th April 2017 to 24th March 2022. The clinical trial included an initial screening and a lead-in phase of seven to ten days, followed by assessments at five time points: baseline, and follow-ups at 6, 12, 24, and 36 weeks. It is important to note that not all measures were collected at each time point, therefore for the current study, longitudinal data could only be utilized from three time points: baseline, and around the 12- and 36-week follow-ups. The primary outcome of the clinical trial was change in depression severity as well as remission and recovery rates (29). The trial has been completed and cross-sectional studies based on its data have been published elsewhere (30–32). As per December 2024, manuscripts related to the primary outcome are undergoing the publication process. The clinical trial was registered at https://www.ClinicalTrials.gov (protocol number NCT03167307). The study was approved by the local ethics committees and was conducted according to the 1964 Declaration of Helsinki and its later amendments. Parents or legal guardians and adolescents aged over 14 years provided written informed consent, younger adolescents and children provided oral assent. The clinical trial was funded by the Swiss National Foundation (grant number 33IC30_166826). No industry funding was received for this study.

### 2.1 Participants

For the current analysis 250 participants aged 8–18 years were included (M = 15.70, SD = 1.60, range 8–18 years, 74% females; see Table 1 for sociodemographic and clinical information). Seven participants were excluded from the initial dataset as they did not complete measurement of HRQoL at any time point. Participants were recruited from seven in- and outpatient centers in four German-speaking cantons of Switzerland. Inclusion criteria were a) diagnosis of MDD according to the Diagnostic and Statistical Manual of Mental Disorders (95), assessed by the Kiddie-Schedule for Affective Disorders Schizophrenia Present and Lifetime (K-SADS) (33), and b) at least a moderate depression severity, defined by reaching a total score of ≥40 in the Children`s Depression Rating Scale revised (96). Exclusion criteria were lifetime diagnosis of schizophrenia, bipolar affective disorder or substance dependency, current eating disorder, mental retardation, developmental disorders (for example autism), clinically relevant somatic disorders and inability to follow study procedures (for example due to insufficient German language skills). Inclusion and exclusion criteria were assessed at screening. All participants received standard treatment of depression according to the German S3 Guidelines for the treatment of depression in children and adolescents (34).

**Table 1:** Sociodemographic and clinical data for depressed adolescents with (*n* = 155) and without (*n* = 83) clinically relevant suicidal ideation (SI) at baseline.

Variables	Total (*n* = 250)	With clinically relevant SI (*n* = 155)	Without clinically relevant SI (*n* = 83)	*p*
*Age* (M (SD) [range])	15.69 (1.60) [8–18]	15.80 (1.35) [11–18]	15.52 (2.03) [8–17]	0.272
*Sex*				0.163
Female	185 (74%)	119 (76.77%)	56 (67.47%)	
Male	65 (26%)	36 (23.23%)	27 (32.53%)	
*Years of education* (M (SD) [range])	8.44 (1.62) [2–12]	8.57 (1.37) [3–12]	8.20 (2.02) [2–12]	0.139
*Highest completed education*				**0**.**013**
Apprenticeship, not yet completed	32 (12.8%)	15 (9.68%)	16 (19.28%)	
High school, not yet completed	49 (19.6%)	32 (20.65%)	14 (16.87%)	
Secondary school, completed	41 (16.4%)	28 (18.06%)	11 (13.25%)	
Secondary school, not yet completed	108 (43.2%)	72 (48.39%)	30 (36.14%)	
*Antidepressant medication*				0.376
Yes	88 (35.2%)	57 (36.77%)	25 (30.12%)	
No	162 (64.8%)	98 (63.23%)	58 (69.88%)	
*Randomization group*				0.356
Placebo	122 (48.8%)	71 (45.81%)	44 (53.01%)	
Omega-3	128 (51.2%)	84 (54,19%)	39 (46.99%)	
*Recurrent episode*				0.670
Yes	55 (22%)	35 (22.58%)	16 (19.285)	
No	195 (78%)	120 (77.42%)	67 (80.72%)	
*Age of onset* (M (SD) [range])	13.87 (2.29) [5–18]	13.94 (2.22) [5–18]	13.84 (2.41) [5–18]	0.747
*Duration of all previous episodes* (M (SD) [range])	15.67 (15.74) [1–156]	14.69 (12.47) [1–87]	16.60 (19.65) [1–156]	0.430
*Suicidal ideation* SIQ-JR Score (M (SD) [range])	40.90 (23.55) [0–89]	55.03 (15.03) [31–89]	14.27 (9.16) [0–30]	**<0.001**
*Depression severity* CDRS-R Score (M (SD) [range])	58.44 (8.63) [41–85]	60.63 (8.34) [42–85]	53.69 (6.92) [41–75]	**<0.001**
*HRQoL* KidScreen-27 Score (M (SD) [range])				
PH	12.86 (3.66) [5–22]	12.29 (3.56) [5–22]	14.07 (3.70) [7–21]	**<0**.**001**
PW	18.03 (5.34) [7–32]	16.19 (4.11) [9–31]	21.78 (5.37) [10–32]	**<0**.**001**
PA	25.74 (5.02) [10–35]	25.54 (4.87) [11–35]	25.85 (5.40) [10–35]	0.666
PE	13.21 (3.62) [4–20]	12.62 (3.64) [4–20]	14.29 (3.39) [4–20]	**<0**.**001**
SC	11.71 (3.10) [4–20]	11.19 (3.00) [4–20]	12.53 (3.14) [4–19]	**0**.**002**

*n* = 12 participants had missing SIQ-JR scores at baseline and could thus not be categorized into one group. SI, suicidal ideation; M, mean; SD, standard deviation; CI, confidence interval; SIQ-JR, Suicidal Ideation Questionnaire Junior; CDRS-R, Children's Depression Rating Scale Revised; HRQoL, health-related quality of life; KidScreen-27 dimensions: PH, physical well-being; PW, psychological well-being; PA, parent relation & autonomy; PE, social support & peers; SC, school environment. All *p*-values refer to *t*-tests expect for sex (Chi-squared test) and highest completed education, marital status and living situation (Fisher's exact test).

Bold values refer to significant *p*-values.

### 2.2 Measures

Sociodemographic information as well as information on antidepressant medication were collected through patients’ medical records as well as through interviews at screening.

Perceived HRQoL was measured using the KidScreen-27 (9, 35). The KidScreen-27 consists of 27 items resulting in five dimensions of HRQoL: physical well-being (PH), psychological well-being (PW), autonomy and parent relation (PA), social support and peers (PE), and school environment (SC). There is a self-report and a parent-report version, each containing similarly phrased items rated on a 5-point Likert scale, asking to evaluate the frequency of a symptom occurring during the last week (35). For this study, only the self-reports and raw values were used in the analyses. All scales show good psychometric properties in various languages, including German (35).

Suicidal ideation was assessed by the Suicidal Ideation Questionnaire Junior (SIQ-JR) (36, 37), a 15-item self-report questionnaire. The instrument provides questions about the frequency of specific suicidal thoughts during the last month, which are rated on a 7-point Likert scale. Reynolds (37) proposed a cut-off value of ≥31 for clinically relevant suicidal thoughts, demanding further assessment from a healthcare professional. The SIQ-JR shows good psychometric properties, also for the German version (36–38).

Depression severity was assessed by the Children's Depression Rating Scale—Revised (CDRS-R) (39), a semi-structured clinical interview assessing 14 depression symptoms rated on a 5- or 7-point Likert scale by patients and parents in separate interviews. The interviewer provides a final score for each symptom. Three non-verbal symptoms are additionally rated solely by the interviewer. The ratings are added to a final score for depression severity; with scores of 0–39 indicating mild, 40–59 indicating moderate and ≥ 60 indicating severe symptom expression. Psychometric properties are good, also for the German version (40, 41).

### 2.3 Statistical analyses

All analyses were conducted in R version 4.3.3 (97). Prior to statistical testing, we assessed the number of missing observations and tested whether missing values were missing at random. At time point 2 and 3, 39 (15.18%) and 85 (33.07%) subjects had dropped out of the study, respectively. However, it is important to note, that some subjects skipped only single questionnaires or single items, resulting in an uneven number of missing values. Thus, we also explored the missing values per questionnaire or subscale of interest and per time point (see Supplementary Table A in the Supplementary Material). Missing data was missing completely at random according to Littlès MCAR test (χ^2^ = 820, df = 850, *p* = 0.763) (42), thus the missing data was omitted from the analyses.

To test our first hypothesis, that HRQoL is lower in depressed adolescents with suicidal ideation than in depressed adolescents without suicidal ideation, we compared the five domains of HRQoL as well as demographic and clinical information between depressed adolescents with clinically relevant suicidal ideation (≥31 in the SIQ-JR) and those without clinically relevant suicidal ideation (< 31 in the SIQ-JR) using either *t*-tests for independent samples (for continuous variables) or Chi-squared or Fisher's exact tests (for categorical variables). Fisher's exact tests were used instead of Chi-squared tests when the assumption of minimum expected frequencies of five was not met (43). The cut-off score of ≥31 for clinically relevant suicidal ideation in the SIQ-JR has been used in previous studies (44, 45).

**Figure 1:**
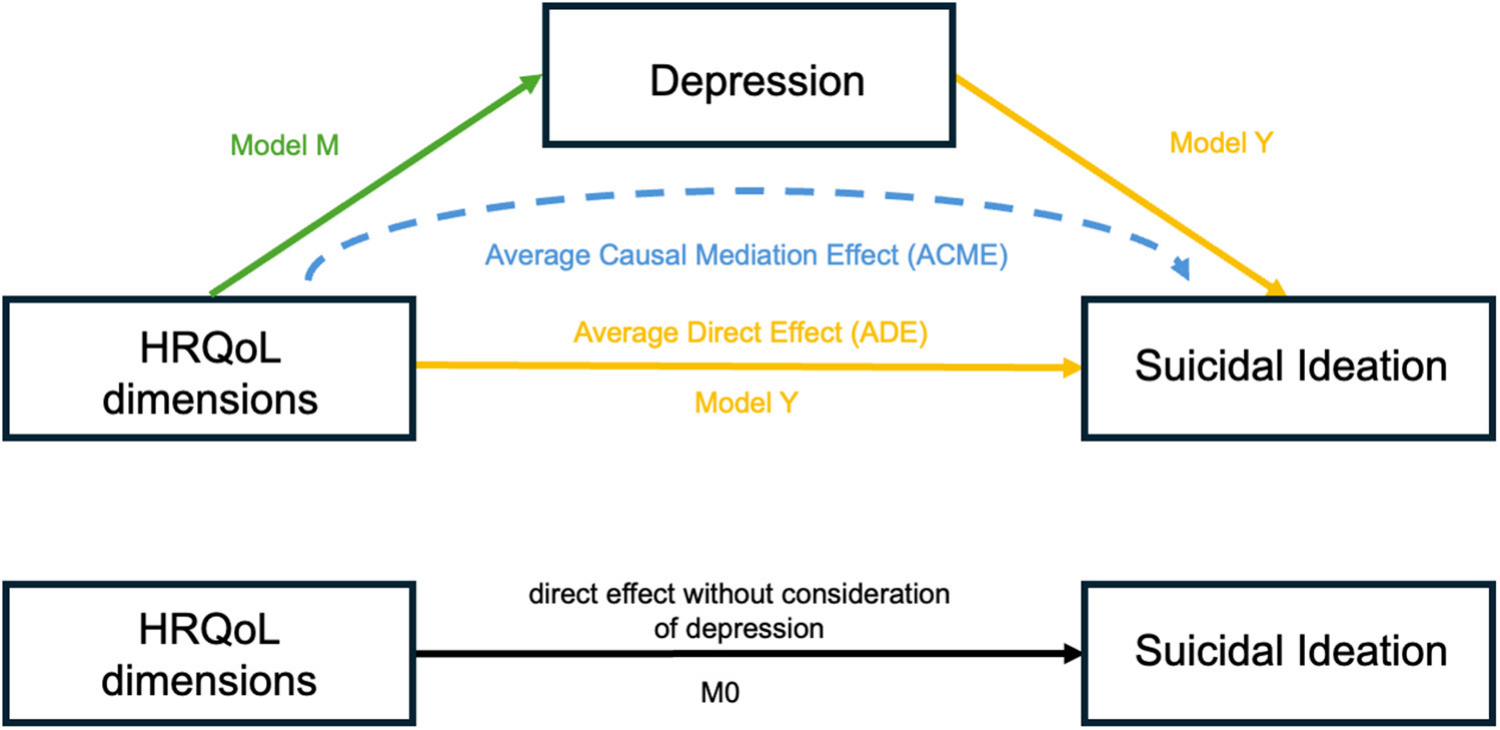
HRQoL, health-related quality of life. Model M0 shows the direct effect of HRQoL dimensions on suicidal ideation without consideration of depression. Model M reflects the effect of HRQoL dimensions on depression. Model Y shows the average direct effect (ADE) of HRQoL dimensions on suicidal ideation with consideration of depression. The indirect effect of HRQoL dimensions on suicidal ideation through the mediation of depression is the average causal mediation effect (ACME).

Next, we used a mediation analysis to investigate the second hypothesis, whether the effect of HRQoL dimensions (PH, PW, PA, PE, SC) on suicidal ideation is mediated by depression severity. However, using the DHARMa package (46), we identified slight violations of the normality assumptions in our linear mixed-effects models. To address this, we employed robust linear mixed-effects models implemented in the “robustlmm” package (47), which are specifically designed to provide reliable parameter estimates in the presence of deviations from normality and other potential model misspecifications. These models are less sensitive to outliers and non-normal residuals, making them a robust alternative for our analyses. Subsequently, we customized the “mediation” package (48) to accommodate the use of robust linear mixed-effects models. In the approach applied by the “robustlmm” package (47), Satterthwaite degrees of freedom are employed to compute *p*-values, which accounts for the robustness of the linear mixed-effects models against potential violations of assumptions such as normality and heteroscedasticity (47, 49). The total effect model (M0) assessed the direct effect of HRQoL dimensions on suicidal ideation disregarding the effect of depression. The mediator model (M) assessed the effect of HRQoL dimensions on depression. The dependent variable model (Y) included the effect of HRQoL dimensions as well as depression on suicidal ideation. All three models included the covariates age, gender and randomization group (Omega-3 or Placebo) as well as a random intercept of subject and a random slope of time (baseline, approximately 12 weeks, and approximately 36 weeks), enabling examination of individual trajectories over time. To account for variations in the timing of follow-up assessments, we used the number of days since baseline as a more precise measure rather than relying on fixed 12- and 36-week intervals. However, given that this variable was on a considerably larger scale, we normalized it by dividing each value by its standard deviation. Models M and Y were used as input for mediation analysis. The indirect effect for each HRQoL dimension was computed as the product of the beta coefficient of the HRQoL dimension from the mediator model (M) and the beta coefficient of the mediator (depression) from the dependent variable model (Y). To compute 95% confidence intervals and *p*-values for the indirect effects, we performed a bootstrap resampling with 500 iterations. Each bootstrap sample involved randomly sampling participants with replacement, refitting the models, and calculating indirect effects. Prior to the mediation analysis, we confirmed a main effect of HRQoL dimensions in either model M0 or M, which is a premise for consecutive mediation analysis (50, 51). Figure 1 shows the proposed mediation relationship. A *p*-value of less than 0.05 was considered statistically significant across all analyses.

To explore potential bidirectional associations, we conducted additional exploratory analyses. Specifically, we computed mediation models applying the same statistical approach as described above, but with depression severity as the predictor and HRQoL dimensions as mediators.

## 3 Results

### 3.1 Descriptive statistics

Table 1 shows sociodemographic and clinical information for the depressed adolescents with and without clinically relevant suicidal ideation at baseline. Statistically significant group differences were found for suicidal ideation (t = −25.94, *p* < 0.001), depression severity (t = −6.86, *p* < 0.001), PH (t = 3.40, *p* = <0.001), PW (t = 8.11, *p* = <0.001), PE (t = 3.47, *p* = <0.001) and SC (t = 3.11, *p* = 0.002), indicating that adolescents with suicidal ideation have a higher depression severity, more suicidal ideation and lower values in PH, PW, PE and SC than adolescents without suicidal ideation. Further, the Fisher's exact test (*p* = 0.013) indicated a significant association between group (with vs. without suicidal ideation) and highest completed education.

### 3.2 Mediating effects of depression on suicidal ideation

To assess whether the effect of HRQoL dimensions on suicidal ideation is mediated by depression, we conducted a mediation analysis. The estimates of the three robust linear mixed effect models M0, M and Y can be found in Tables 2–4, respectively. For the total effect model M0 (Table 2), we found a statistically significant negative effect of PW (*β* = −1.89, *p* < 0.001) on suicidal ideation, suggesting that improvement of psychological well-being leads to reduced suicidal ideation. All other dimensions of HRQoL (PH, PA, PE, SC) as well as the covariates age, gender and randomization group did not reach statistical significance. Additionally, the model revealed a statistically significant negative effect of time (*β* = −3.20, *p* < 0.001) on suicidal ideation, indicating a decrease of suicidal ideation over time.

**Table 2:** Total effect model M0: robust linear mixed effect model showing the effect of HRQoL dimensions on suicidal ideation.

Predictors	Suicidal Ideation				
	β	SE	CI	t-value	*p*-value
(Intercept)	75.51	12.25	51.50–99.51	6.17	**<0.001**
Time point	−3.20	0.81	−4.78–1.61	−3.96	**<0.001**
PH	0.24	0.24	−0.22–0.70	1.01	0.311
PW	−1.89	0.17	−2.23–1.55	−10.88	**<0.001**
PA	−0.02	0.17	−0.34–0.31	−0.10	0.920
PE	−0.02	0.21	−0.44–0.40	−0.09	0.927
SC	0.13	0.23	−0.33–0.59	0.57	0.570
Age	−0.46	0.68	−1.78–0.87	−0.68	0.496
Sex (female)	3.85	2.50	−1.06–8.76	1.54	0.124
Randomization group (placebo)	−2.48	2.14	−6.69–1.73	−1.16	0.247
Random effects	Variance	SD			
Subject	298.88	17.29			
Time point	11.96	3.46			
Residual	113.22	10.64			
Goodness of fit	R^2^	Conditional R^2^			
	0.31	0.77			

This table shows the direct effect of HRQoL dimensions on suicidal ideation disregarding the effect of depression. The model includes the covariates age, sex, and randomization group, as well as a random intercept of subject and a random slope of time. SE, standard error; SD, standard deviation; CI, confidence interval; KidScreen-27 dimensions: PH, physical well-being; PW, psychological well-being; PA, parent relation & autonomy; PE, social support & peers; SC, school environment.

Bold values refer to significant *p*-values.

**Table 3:** Mediator model M: robust linear mixed effect model showing the effect of HRQoL dimensions on depression.

Predictors	Depression				
	β	SE	CI	t-value	*p*-value
(Intercept)	75.26	4.97	65.52–85.01	15.14	**<0.001**
Time point	−7.63	0.55	−8.72−6.54	−13.76	**<0.001**
PH	−0.33	0.12	−0.58−0.09	−2.69	**0.007**
PW	−0.65	0.09	−0.83−0.47	−7.05	**<0.001**
PA	−0.12	0.08	−0.28–0.04	−1.50	0.133
PE	−0.24	0.11	−0.47−0.02	−2.12	**0.034**
SC	−0.27	0.13	−0.53−0.02	−2.11	**0.035**
Age	0.20	0.26	−0.31–0.71	0.78	0.439
Sex (female)	1.00	0.97	−0.91–2.90	1.03	0.305
Randomization group (placebo)	−0.45	0.81	−2.03–1.14	−0.55	0.580
Random effects	Variance	SD			
Subject	4.00	2.00			
Time point	7.99	2.83			
Residual	50.69	7.12			
Goodness of fit	R^2^	Conditional R^2^			
	0.57	0.69			

This table shows the effect of HRQoL dimensions on depression. The model includes the covariates age, sex, and randomization group, as well as a random intercept of subject and a random slope of time. SE, standard error; SD, standard deviation; CI, confidence interval; KidScreen-27 dimensions: PH, physical well-being; PW, psychological well-being; PA, parent relation & autonomy; PE, social support & peers; SC, school environment.

Bold values refer to significant *p*-values.

**Table 4:** Dependent Variable model Y: robust linear mixed effect model showing the effect of HRQoL dimensions and depression on suicidal ideation.

Predictors	Suicidal Ideation				
	β	SE	CI	t-value	*p*-value
(Intercept)	43.03	12.93	17.70–68.37	3.33	**0.001**
Time point	0.18	0.97	−1.72–2.07	0.18	0.856
Depression	0.44	0.08	0.29–0.59	5.83	**<0.001**
PH	0.39	0.23	−0.06–0.84	1.72	0.085
PW	−1.61	0.18	−1.96−1.27	−9.21	**<0.001**
PA	0.05	0.16	−0.27–0.36	0.28	0.779
PE	0.03	0.21	−0.38–0.44	0.14	0.829
SC	0.25	0.23	−0.20–0.70	1.10	0.272
Age	−0.53	0.64	−1.78–0.71	−0.84	0.402
Sex (female)	3.33	2.36	−1.30–7.95	1.41	0.158
Randomization group (placebo)	−2.23	2.02	−6.19–1.73	−1.10	0.270
Random effects	Variance	SD			
Subject	287.38	16.95			
Time point	16.24	4.03			
Residual	107.24	10.36			
Goodness of fit	R^2^	Conditional R^2^			
	0.35	0.78			

This table shows the effect of depression and HRQoL dimensions on suicidal ideation. The model includes the covariates age, sex, and randomization group, as well as a random intercept of subject and a random slope of time. SE, standard error; SD, standard deviation; CI, confidence interval; KidScreen-27 dimensions: PH, physical well-being; PW, psychological well-being; PA, parent relation & autonomy; PE, social support & peers; SC, school environment.

Bold values refer to significant *p*-values.

For the mediator model M (Table 3), we found statistically significant negative effects for PH (*β* = −0.33, *p* = 0.007), PW (*β* = −0.65, *p* < 0.001), PE (*β* = −0.24, *p* = 0.034) and SC (*β* = −0.27, *p* = 0.035) on depression, indicating that an improvement in these dimensions of HRQoL is associated with lower depression severity. PA as well as the covariates age, gender and randomization group did not yield statistically significant effects. Additionally, the model showed a statistically significant negative effect of time (*β* = −7.63, *p* < 0.001) on depression, indicating a decrease of depression over time.

For the dependent variable model Y (Table 4), we found a statistically significant negative effect for PW (*β* = −1.61, *p* < 0.001) on suicidal ideation, indicating that increased psychological well-being is associated with lower suicidal ideation when taking depression severity into consideration (average direct effect; ADE). The model also showed a statistically significant positive effect of depression (*β* = 0.44, *p* < 0.001) on suicidal ideation, suggesting that increased depression is associated with increased suicidal ideation. The other dimensions of HRQoL (PH, PA, PE, SC) as well as time and the covariates age, gender and randomization group did not reach statistical significance.

For all three models M0, M and Y we observed a substantial variation in the baseline levels of suicidal ideation among subjects, as indicated by the random intercept (SD = 17.29, 2.00 and 16.95 for models M0, M and Y, respectively), and a considerable variability in the rate of change over time of suicidal ideation across subjects, as indicated by the random slope (SD = 3.46, 2.83 and 4.03 for models M0, M and Y, respectively).

**Figure 2:**
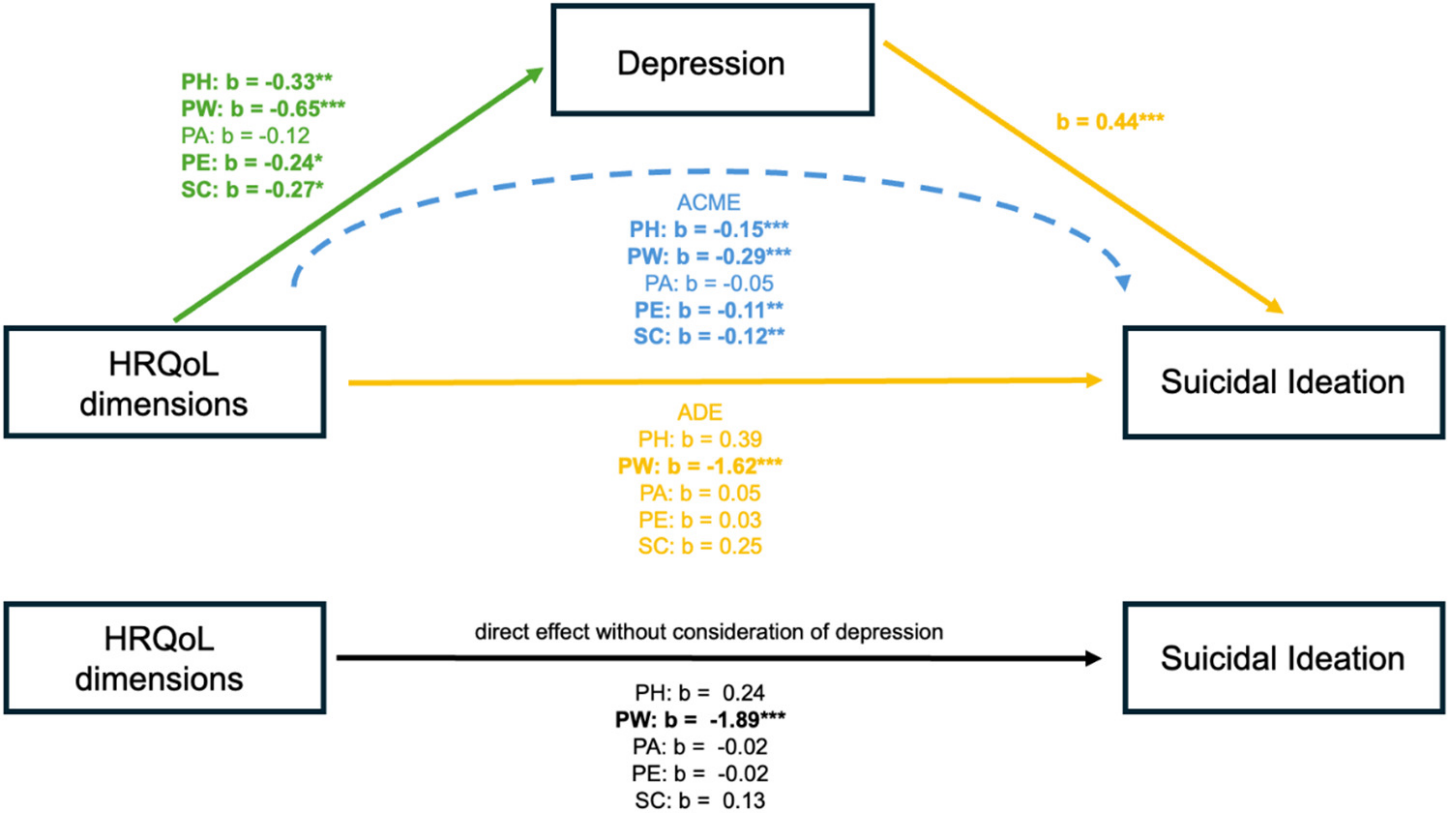
HRQoL, health-related quality of life; KidsScreen-27 dimensions: PH, physical well-being; PW, psychological well-being; PA, parent relation & autonomy; PE, social support & peers; SC, school environment; ADE, average direct effect; ACME, average causal mediation effect; ***p < 0.001; **p < 0.01; *p < 0.05.

Finally, we computed the average causal mediation effect (ACME) of HRQoL dimensions on suicidal ideation. This procedure yielded a statistically significant ACME of PH (*β* = −0.15, *p* < 0.001), PW (*β* = −0.29, *p* < 0.001), PE (*β* = −0.11, *p* = 0.004) and SC (*β* = −0.12, *p* = 0.008) on suicidal ideation, suggesting that depression severity mediates the effect of these dimensions of HRQoL on suicidal ideation. The effect of PA did not reach statistical significance. The results of the mediation analysis are summarized in Figure 2.

The additional exploratory analyses indicate that the HRQoL dimensions PH (ACME: *β* = 0.08, *p* < 0.001), PW (ACME: *β* = 0.42, *p* < 0.001), PA (ACME: *β* = 0.03, *p* = 0.028), PE (ACME: *β* = 0.05, *p* < 0.001), and SC (ACME: *β* = 0.05, *p* < 0.001) serve as mediators in the relationship between depression severity and suicidal ideation. See Supplementary Figures A–E in the Supplementary Material for the results of these additional exploratory analyses.

## 4 Discussion

In this study, we investigated the relationship of health-related quality of life (HRQoL), suicidal ideation and depression utilizing longitudinal data of 250 children and adolescents diagnosed with MDD. At baseline, we found that depressed adolescents with suicidal ideation showed significantly higher depression severity and lower HRQoL in four out of five dimensions—physical well-being (PH), psychological well-being (PW), social support and peers (PE), and school environment (SC)—compared to those without suicidal ideation. Our mediation analysis revealed that the relationship between HRQoL dimensions and suicidal ideation was mediated by depression severity. While only the PW dimension showed a statistically significant direct effect on suicidal ideation, the mediation analysis indicated that PH, PW, PE, and SC had significant indirect effects, meaning their impact on suicidal ideation was mediated through depression severity. These findings highlight the importance of addressing both HRQoL and depression in interventions targeting suicidality in adolescents with MDD.

### 4.1 Differences in depression severity and health-related quality of life at baseline

At baseline, the observed group differences between adolescents with and without suicidal ideation emphasize the substantial impact of suicidal ideation on both depression severity and various dimensions of HRQoL. These findings are consistent with existing literature indicating a robust association between suicidal ideation and increased depression severity in both general and adolescents populations (52, 53). Additionally, we observed significant group differences in HRQoL dimensions (PH, PW, PE and SC), indicating that adolescents with suicidal ideation have lower HRQoL in these domains than those without suicidal ideation. This is in line with previous research demonstrating an inverse relationship between HRQoL and suicidality as well as self-harm in both adult and adolescent populations (11, 16, 54, 55).

### 4.2 Longitudinal changes in depression severity and suicidal ideation

The analysis of longitudinal data indicated a decline in both depression severity and suicidal ideation over time, consistent with the fact that participants were receiving standard treatment for depression. This aligns with the German S3 Guidelines for the treatment of depression in children and adolescents, indicating that therapeutic interventions, such as cognitive-behavioral therapy, medication, or a combination of both, can lead to significant reductions of depressive symptoms and suicidal ideation over time (34). However, there was a substantial variability in baseline levels of suicidal ideation and depression across participants, as well as in the rates of change over time, which indicates that individual trajectories differ considerably, highlighting the need for personalized approaches to treatment. These findings align with existing longitudinal studies, which have shown that depression and suicidal ideation often improve with appropriate interventions, but that the degree and speed of recovery can vary (56, 57). Overall, the observed decrease in depression and suicidal ideation over time highlight the effectiveness of standard treatments in alleviating these issues, while also emphasizing the importance of monitoring individual progress and adjusting interventions as needed to ensure long-term recovery.

### 4.3 The mediating role of depression severity in the relationship between health-related quality of life and suicidal ideation

The longitudinal analysis of HRQoL dimensions revealed that only psychological well-being had a direct impact on suicidal ideation. This is in line with previous research, such as the prospective study with 573 young individuals by Russell et al. (58), which demonstrated an association between mental well-being and later self-harming thoughts and behaviors, likely mediated by feelings of defeat and entrapment. Similarly, Morey et al. (59) showed that lower levels of mental well-being were associated with self-harming behavior in a cross-sectional sample of 2’000 adolescents. However, in our analysis, psychological well-being influenced suicidal ideation both directly and indirectly through its effect on depression severity. This finding is unsurprising, as psychological well-being is closely linked to depression, with psychological well-being influencing subsequent depression and vice versa in a population of adolescents (60). Other studies have identified similar mediating effects for depression severity or related constructs. For instance, Chukwuemeka et al. (19) demonstrated that psychological distress mediated the effect of HRQoL on suicidal ideation in adult cancer patients in Nigeria.

In contrast, the other HRQoL dimensions (PH, PA, PE, and SC) did not exhibit a direct effect on suicidal ideation. However, depression severity mediated the impact of PH, PE, and SC on suicidal ideation. This is consistent with a substantial body of literature regarding the association between physical activity, depression and suicidality. Numerous recent systematic reviews and meta-analyses in adult populations indicate a significant association between physical activity and reduced depressive symptoms (61–63). For example, Pearce et al. (62) found that adults meeting the recommended physical activity guidelines (2.5 h of walking per week or equivalent) had a 25% reduced risk of depression. Sibold et al. (64) revealed that students engaging in physical activity four to five days per week were less likely to exhibit suicidal ideation than students engaging in physical activity on only one or no day a week. Further, several studies support our finding that depressed adolescents with suicidal ideation report lower physical well-being compared to those without suicidal ideation. For instance, Gyori et al. (54) observed lower physical HRQoL in adolescents engaging in self-harming behavior, while Li et al. (5) found lower physical HRQoL in students with depression compared to their peers without depression. Those associations can be attributed to several neurophysiological, psychological and social mechanisms, including neuroendocrine and inflammatory responses, as well as improved body image, all of which are directly related to higher physical well-being (62, 63). Other studies, such as those by Algorta et al. (65) and Fairweather-Schmidt et al. (11), reported no significant effects of physical well-being on suicidal ideation, and in some cases, young adults with suicidal ideation exhibited better physical HRQoL than those without. These discrepancies may stem from methodological differences and the lack of consensus on the dimensions of HRQoL and their operationalization.

Our findings further suggest that peer relationships influence suicidal ideation indirectly by affecting depression severity, aligning with literature highlighting the protective role of social support for various psychological outcomes. Social support is an established protective factor against depression (66) and suicidality (67, 68). However, some studies have reported contradictory findings, indicating no significant association (69) or a relationship only for support from family members, rather than peers (70, 71). Wan et al. (72) found depressive symptoms to mediate between perceived social support and suicidal ideation in an adolescent population in China. Additionally, negative peer interactions, such as bullying, can exacerbate depression and increase the risk of suicidal ideation. Gómez-Tabares (21) showed that depression mediated the link between bullying and suicidal behavior. Bullying, which is often assessed in a school environment, has been consistently linked to both depressive symptoms (73, 74) as well as suicidality (75–77), suggesting that the social environment at school plays a critical role in influencing both depression and suicidal ideation. Other aspects of school environment in varying cultural contexts are also associated with suicidal ideation, including school safety and the availability of illegal drugs, as demonstrated in the study by Pfledderer et al. (78) in the USA, and stressful psychosocial school environment, measured by effort-reward imbalance, in a Chinese population experiencing suicidal ideation (79).

Unexpectedly, our analysis did not reveal any significant group differences in the parent relation and autonomy dimension of HRQoL (PA) between depressed adolescents with and without suicidal ideation at baseline, nor did PA significantly contribute to the mediation models. This suggests that there is no effect of PA on suicidal ideation or depression severity and consequently depression severity also does not mediate the effect of PA on suicidal ideation. This result is contrary to our hypotheses and existing literature such as Low (25), who found that depression mediated the effect of parent-adolescent conflict on suicidal ideation. Based on previous literature we would have expected the family and peer domains of HRQoL to be especially relevant in this age group. Several studies show the importance of family factors for suicidality and self-harm, such as quality of interaction with parents (80), perceived family support (98), family dysfunction and absent parents (81). In the systematic review by Le et al. (16) several studies emphasize the importance of family domains of HRQoL in this population. Four studies reported a significant negative association of family related HRQoL with self-harm (54, 55) and suicidal attempt (65, 82). Three studies showed a negative association between family-related HRQoL and suicidal ideation (83–85). Gyori et al. (54) even found the only direct association between HRQoL and self-harm for the family area, which seems to highlight the importance of the family for self-harm.

A possible explanation for our findings might be the developmental shift during adolescence, where peer relationships become more essential than family relationships for social support (86–88). The study by Zullig (55) supports this hypothesis, finding only a weak although significant association between deliberate self-harm and family satisfaction, but the strongest association for satisfaction with friendships in a sample of college students. Another potential explanation could relate to the methodology used to assess the PA dimension in the KidScreen-27 questionnaire (9). The PA dimension includes broad and somewhat vague items that assess not only parent relationships but also autonomy, with questions like ‘Have you had enough time for yourself?’ or ‘Have you been able to do the things you want to do in your free time?’. These items may be influenced by external factors unrelated to the family, such as academic stress. Moreover, the items addressing parent relationships are quite general and do not capture specific family conflicts, which might explain the divergence from findings like those of Low (25). In contrast, other HRQoL dimensions, such as PE, include more precise items about social support. Additionally, the PA dimension appears to be more stable over time, as family relationships tend to be relatively constant during adolescence, making it harder to detect significant changes or effects. This stability aligns with research suggesting that family relationships are less volatile than peer relationships in early adolescence, where friendships are more likely to change frequently (87).

### 4.4 Strengths and limitations

A key strength of the current study is its longitudinal design combined with a large sample size. Furthermore, we employed robust linear mixed-effects models to analyze the data, which are well-suited for longitudinal analyses when not all assumptions for standard linear mixed-effects models are met. However, this approach did not permit the examination of reciprocal relationships. This is significant, as previous studies have demonstrated that HRQoL mediates the relationship between internalizing psychopathology and suicidal ideation in adolescent clinical samples (89). This is in line with the findings of our additional exploratory analyses, showing that HRQoL dimensions mediate the relationship between depression severity and suicidal ideation. The study of Gyori et al. (54) used network modelling to test the association between HRQoL dimensions and non-suicidal self-harm in 202 adolescents. The authors found that, contrary to their initial hypothesis, mental disorders mediated the effect between HRQoL dimensions and non-suicidal self-harm behavior and not the reverse. More recent studies, such as that by Soto-Chavarría et al. (26), also support this proposed relationship. Their findings indicate that depression mediates the association between HRQoL and suicidal ideation in pregnant female adolescents. Our additional exploratory analyses indicate that both depression and HRQoL dimensions may act as mediators. This suggests that depression might deteriorate HRQoL and thereby indirectly increase suicidal ideation. On the other hand, impaired HRQoL may lead to depressive symptoms, which in turn increase suicidal ideation.

Further bidirectional effects between the examined variables are plausible. For instance, prior research suggests that suicidal ideation may lead to lower HRQoL (11, 90). Similarly, while depression is commonly assessed as precursor to suicidality, reverse effects cannot be ruled out (91). Depression severity may also influence participants’ answers in the KidScreen-27, as the dimension regarding psychological well-being includes items assessing mood, listlessness, and self-esteem, which are typical symptoms of depression (9, 35, 39). Structural equation modeling would be a valuable approach to examine such potential bidirectional associations. However, the current sample size was insufficient for these complex analyses, increasing the risk of poor model fit and convergence problems (92). Therefore, further studies employing even larger samples and a longitudinal design, utilizing advanced statistical methods such as cross-lagged panel models, may be necessary to more thoroughly investigate potential reciprocal relationships between HRQoL, depression and suicidality.

Furthermore, there was a substantial amount of missing data at follow-up, which may affect the reliability and validity of the findings due to attrition bias. Attrition bias arises when participants who drop out differ systematically from the remaining study sample (93). In our study, individuals with more severe symptoms may have been more likely to discontinue participation—potentially due to low motivation, a common feature of severe depression—which could have led to an underestimation of symptom severity at follow-up (94). Therefore, our results regarding the longitudinal decline of depression severity and suicidal ideation should be interpreted with caution.

Additionally, the generalizability of the results is limited, as the study focused on a specific clinical sample of depressed adolescents. Future studies should be conducted in more heterogeneous samples, as HRQoL may be more effective in distinguishing individuals with lower symptomatology (10). Moreover, future research should not only examine suicidal ideation but also suicidal behaviors, exploring the distinct impact of HRQoL on both. Further investigations into the individual effects of different HRQoL dimensions are also warranted.

### 4.5 Conclusion and practical implications

In conclusion, this study provides insight into the complex relationship of HRQoL, depression and suicidal ideation in a clinical sample of adolescents. Our findings show that while psychological well-being had a direct effect on suicidal ideation, other HRQoL dimensions, such as physical well-being, social support and peers, and school environment, influenced suicidal ideation indirectly through depression severity. Clinically, these findings support the use of HRQoL assessments as a tool for identifying suicide risk and emphasize the need for resource-oriented therapeutic approaches. Rather than focusing solely on diagnostic criteria, interventions should target HRQoL domains to extend support beyond therapy, improving outcomes in real-life settings like schools. By addressing broader aspects of adolescents’ well-being, such interventions can potentially reduce depression and prevent suicide in this vulnerable population more effectively.

## Data Availability

The raw data supporting the conclusions of this article will be made available by the authors, without undue reservation.
