# Supplementary material for: The mediating role of depression in the association between health-related quality of life and suicidal ideation in adolescents: findings from a longitudinal study
Source: Front Child Adolesc Psychiatry. 2025 Aug 14;4:1567387. doi: 10.3389/frcha.2025.1567387 (PMC12390987; doi:10.3389/frcha.2025.1567387)
Supplement: Supplementary file 1 [file Datasheet1.pdf]

Supplementary Material

Table A

Missing values per questionnaire and subscale per time-point

	**Time-points**		
	1	***2***	***3***
SIQ-JR	12 (4.8%)	54 (21.6%)	105 (42%)
CDRS-R	0 (0%)	37 (14.8%)	79 (31.6%)
HRQoL			
PH	26 (10.4%)	65 (26%)	107 (42.8%)
PW	11 (4.4%)	60 (24%)	108 (43.2%)
PA	8 (3.2%)	61 (24.4%)	106 (42.4%)
PE	8 (3.2%)	59 (23.6%)	105 (42%)
SC	12 (4.8%)	63 (25.2%)	110 (44%)

Note: SIQ-JR, Suicidal Ideation Questionnaire Junior; CDRS-R, Children's Depression Rating Scale - Revised; HRQoL, health related quality of life; KidScreen-27 dimensions: PH, physical well-being; PW, psychological well-being; PA, parent relation & autonomy; PE, social support & peers; SC, school environment.

Figure A

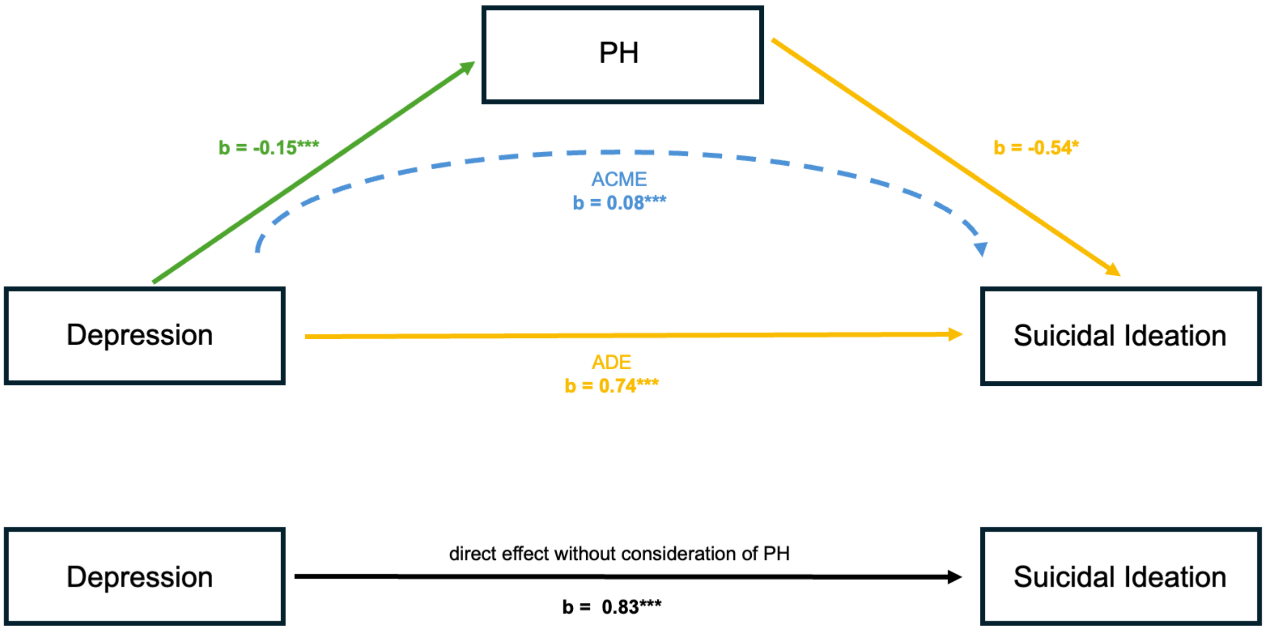

Figure A: Mediation model showing the relationship between depression and suicidal ideation, as well as the mediation of this relationship by the HRQoL dimension PH. The mediation model is based on robust linear mixed effect models. The models include the covariates age, sex, and randomization group, as well as a random intercept of subject and a random slope of time. PH, physical well-being dimension of the KidScreen-27 questionnaire; ADE = average direct effect; ACME = average causal mediation effect; *** = $p < 0.001$; ** = $p < 0.01$; * = $p < 0.05$.

Figure B

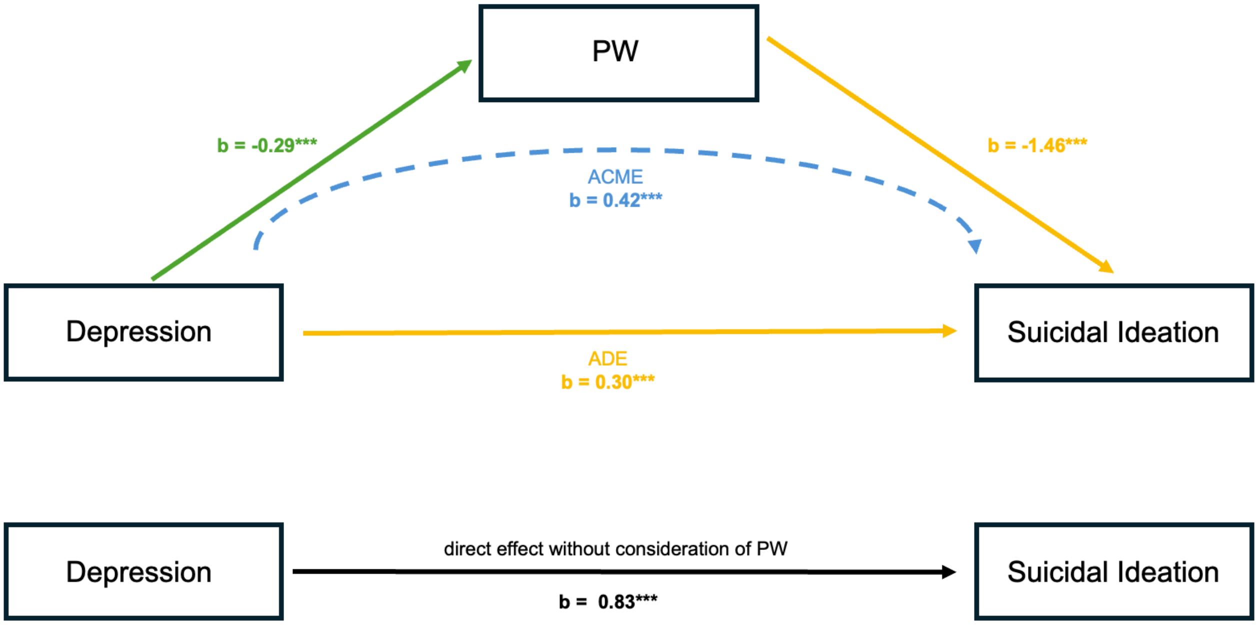

Figure B: Mediation model showing the relationship between depression and suicidal ideation, as well as the mediation of this relationship by the HRQoL dimension PW. The mediation model is based on robust linear mixed effect models. The models include the covariates age, sex, and randomization group, as well as a random intercept of subject and a random slope of time. PW, psychological well-being dimension of the KidScreen-27 questionnaire; ADE = average direct effect; ACME = average causal mediation effect; *** = $p < 0.001$; ** = $p < 0.01$; * = $p < 0.05$.

Figure C

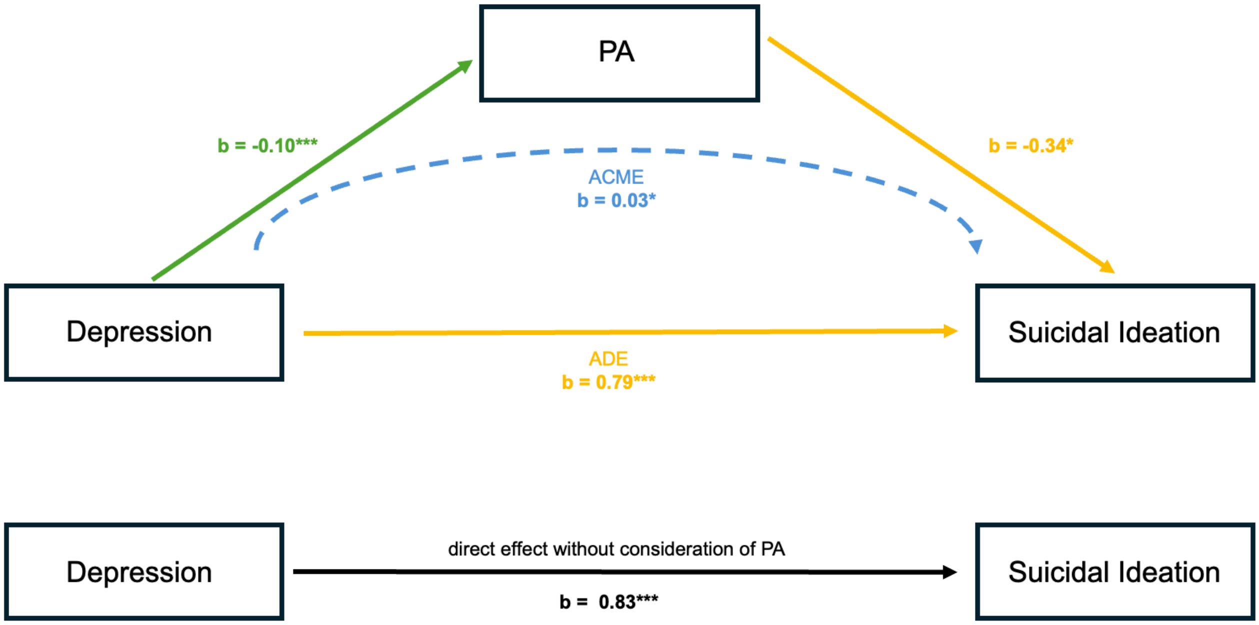

Figure C: Mediation model showing the relationship between depression and suicidal ideation, as well as the mediation of this relationship by the HRQoL dimension PA. The mediation model is based on robust linear mixed effect models. The models include the covariates age, sex, and randomization group, as well as a random intercept of subject and a random slope of time. PA, parent relation & autonomy dimension of the KidScreen-27 questionnaire; ADE = average direct effect; ACME = average causal mediation effect; *** = p < 0.001; ** = p < 0.01; * = p < 0.05.

Figure D

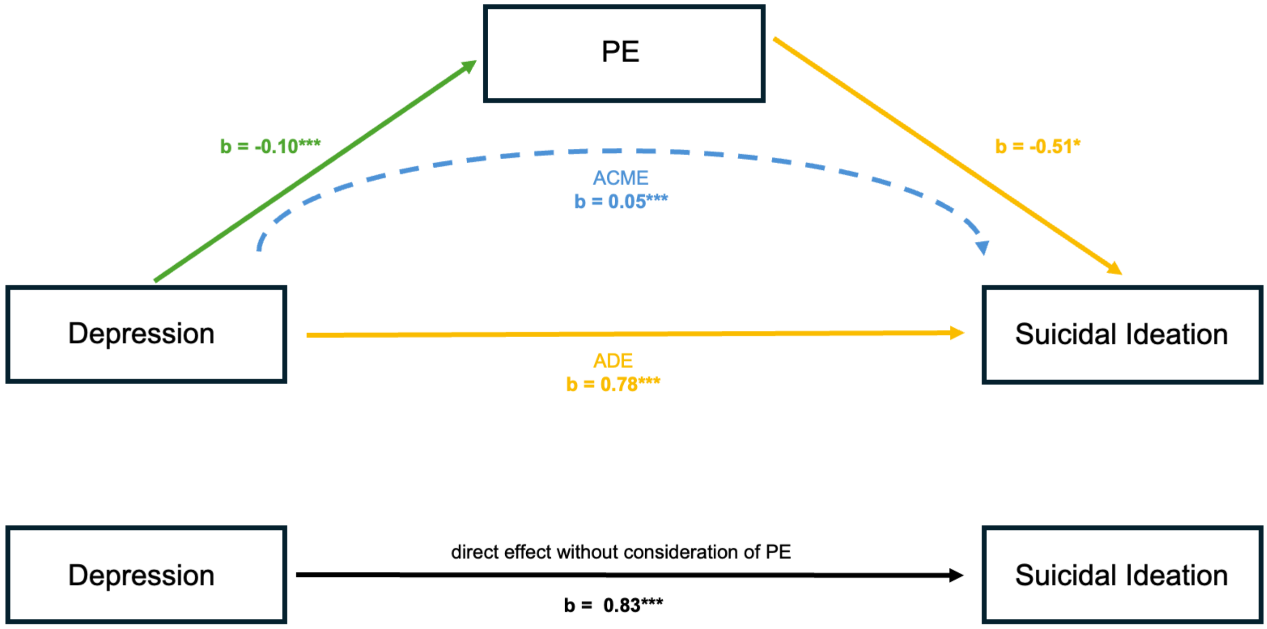

Figure D: Mediation model showing the relationship between depression and suicidal ideation, as well as the mediation of this relationship by the HRQoL dimension PE. The mediation model is based on robust linear mixed effect models. The models include the covariates age, sex, and randomization group, as well as a random intercept of subject and a random slope of time. PE, social support & peer dimension of the KidScreen-27 questionnaire; ADE = average direct effect; ACME = average causal mediation effect; *** = $p < 0.001$; ** = $p < 0.01$; * = $p < 0.05$.

Figure E

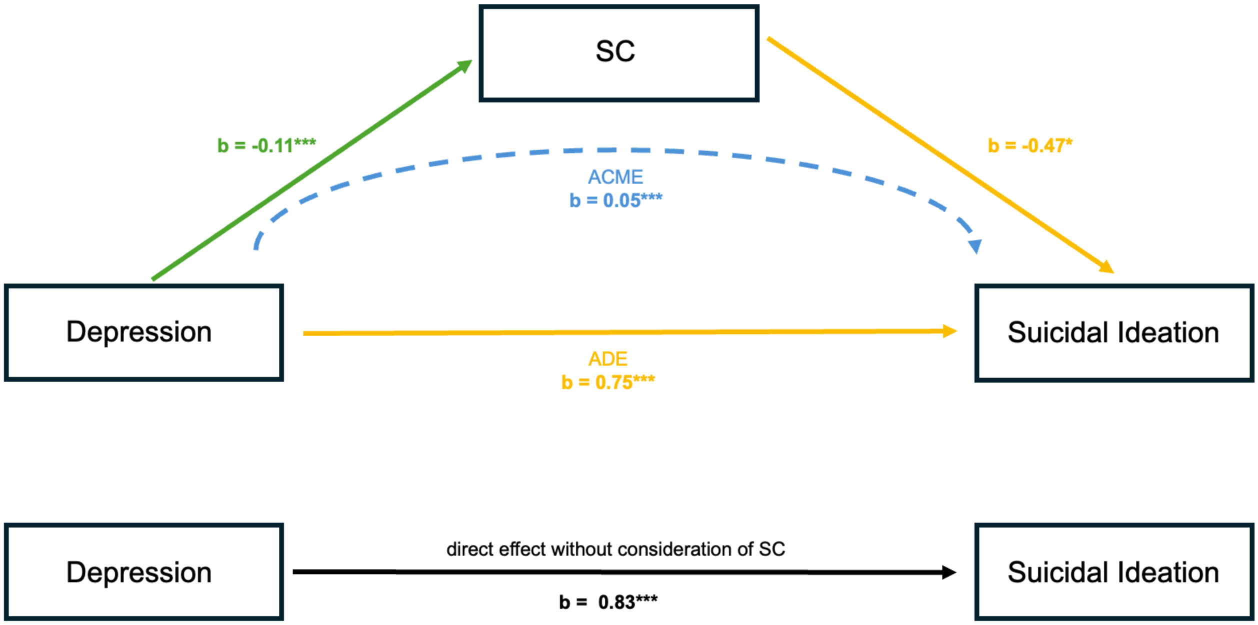

Figure E: Mediation model showing the relationship between depression and suicidal ideation, as well as the mediation of this relationship by the HRQoL dimension SC. The mediation model is based on robust linear mixed effect models. The models include the covariates age, sex, and randomization group, as well as a random intercept of subject and a random slope of time. SC, school environment dimension of the KidScreen-27 questionnaire; ADE = average direct effect; ACME = average causal mediation effect; *** = $p < 0.001$; ** = $p < 0.01$; * = $p < 0.05$.
